# The complex evolutionary history and phylogeography of *Caridina typus* (Crustacea: Decapoda): long-distance dispersal and cryptic allopatric species

**DOI:** 10.1038/s41598-017-08494-w

**Published:** 2017-08-22

**Authors:** Samuel C. Bernardes, Almir R. Pepato, Thomas von Rintelen, Kristina von Rintelen, Timothy J. Page, Hendrik Freitag, Mark de Bruyn

**Affiliations:** 1 Laboratório de Acarologia, Instituto de Ciências Biológicas, Universidade Federal de Minas Gerais, Av. Antônio Carlos 6627, Belo Horizonte, 31270-901 Brazil; 2 Museum für Naturkunde, Leibniz Institute for Evolution and Biodiversity Science, Invalidenstraße 43, Berlin, 10115 Germany; 3 Australian Rivers Institute, Griffith University, Nathan, Queensland 4111, Australia; 4 Water Planning Ecology, Queensland Department of Science, Information Technology and Innovation, Dutton Park, Queensland 4102, Australia; 5 Department of Biology, School of Science & Engineering, Ateneo de Manila University, Quezon City, 1108 Philippines; 6 School of Biological Sciences, Bangor University, Bangor, LL57 2UW UK; 7 School of Life and Environmental Sciences, The University of Sydney, Sydney, NSW 2006 Australia

## Abstract

The evolutionary history of the old, diverse freshwater shrimp genus *Caridina* is still poorly understood, despite its vast distribution – from Africa to Polynesia. Here, we used nuclear and mitochondrial DNA to infer the phylogeographic and evolutionary history of *C*. *typus*, which is one of only four species distributed across the entire range of the genus. Despite this species’ potential for high levels of gene flow, questions have been raised regarding its phylogeographic structure and taxonomic status. We identified three distinct lineages that likely diverged in the Miocene. Molecular dating and ancestral range reconstructions are congruent with *C*. *typus*’ early dispersal to Africa, possibly mediated by the Miocene Indian Ocean Equatorial Jet, followed by back dispersal to Australasia after the Jet’s closure. Furthermore, several different species delimitation methods indicate each lineage represents a distinct (cryptic) species, contradicting current morphospecies delimitation of a single *C*. *typus* taxon. The evolutionary history of *C*. *typus* lineages is complex, in which ancient oceanic current systems and (currently unrecognised) speciation events preceded secondary sympatry of these cryptic species.

## Introduction

The Atyidae (Crustacea: Decapoda: Caridea) is a family of small freshwater shrimps that occurs across all continents, except Antarctica. Fossil and molecular data show that the atyids are an old taxon, with estimates of divergence from other carideans ranging from the Permian to the Cretaceous^1, 2^. The most speciose and most widely distributed atyid genus is *Caridina*, with over 300 species^3^ found from Africa to the Pacific islands, a range closely congruent with that of its type species, *Caridina typus* H. Milne-Edwards, 1837. *Caridina* have been shown to be highly tolerant to high salinity levels, even in larval stages^4^, and *C*. *typus* is never found too far from the sea^5, 6^, which could help to explain its wide distributional range. Small eggs and planktonic larvae^4^ are also a feature that could contribute to *C*. *typus*’ vagility^7^. Despite this widespread distribution, *C*. *typus*, like other species of *Caridina* ^8–10^, may actually comprise cryptic lineages. Phylogeographic and demographic studies on *C*. *typus* are limited, however Page *et al*.^11^ recovered strong support for a shallow intraspecific clade containing widely distributed *C*. *typus* specimens from Australia, Sri Lanka and several Pacific islands. A similar pattern of shared haplotypes was identified among populations of atyids isolated on distant islands of the Caribbean^12^. However, the opposite pattern has also been found for several recently identified (previously) cryptic species of *Caridina* ^8, 13, 14^, where deep intraspecific differences have been found over very small scales. In fact, these cryptic species displayed differences that could not only serve as an identifiable character^8^ but also as a rough indicator of dispersal ability and consequent phylogeographic structure^15^.

*Caridina typus*’ distribution is interesting because it comprises several areas with a very active geological history. First, the species is found in the Seychelles and in the Mascarene Islands, both part of the Mascarene Plateau: the former are granitic islands, fragments of Gondwana, whereas the latter are much younger volcanic islands^16, 17^. *C*. *typus* is also found through the Indo-Australian Archipelago (hereafter IAA), a region with a rich geological and biogeographical history. The IAA was formed and shaped by the movement of the Australian Plate against the Sunda Shelf, and much of this region has been cyclically submerged and exposed through the Cenozoic, as the Earth’s ice-sheets have waxed and waned. Although thousands of islands arose from the Australian Plate’s northward movement, many islands represented landmasses that were previously connected to Eurasia^18^. Dispersal seems to be a very important process in this area, since most of the islands have never been connected to other land areas^19^, but vicariant events may also have been of importance with the opening of marine seaways, and orogeny that turned the landscape from a wide peninsula into the Archipelago we see today^20, 21^.

The history of the IAA has also influenced the biogeography of the Indian and Pacific Oceans. Before Sulawesi was formed, both oceans were widely interconnected, but Gourlan *et al*.^22^ found evidence to support the existence of the so called ‘Miocene Indian Ocean Equatorial Jet’ (hereafter MIOJet), a strong westward current that would have restricted eastward transport. This current persisted until Sulawesi was completely established as a single landmass, an event that initiated the currents we see today, and limited exchange between the two Oceans in the equatorial zone via the Indonesian Throughflow.

**Figure 1:**
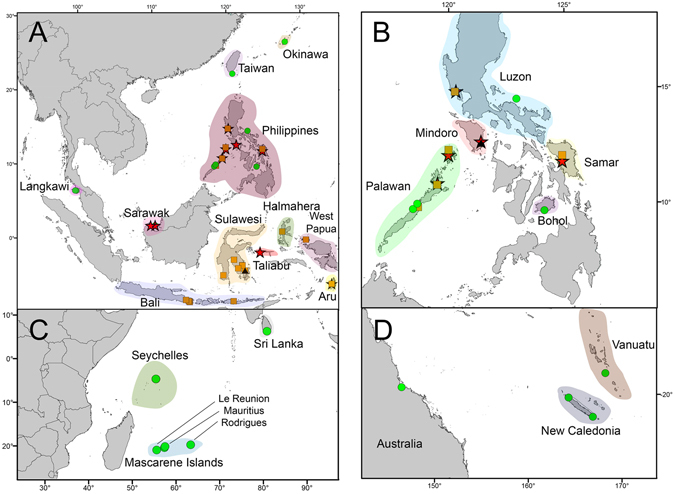
Sampling map of the Indo-Australian Archipelago (IAA) and Eastern Asia (**a**), with detail for Philippine islands (**b**); Western Indian Ocean (**c**) and South Pacific (**d**). The colours represent the regions and the symbols illustrate the three major clades (putative species): stars represent TAL, circles represent ARC and squares represent SUL. The triangles represent the other *Caridina* samples, *C*. *villadolidi* and *C*. *opaensis*. Map and georeferencing were generated through ArcGIS Desktop: Release 10.

Given its broad distribution, populations attributed morphologically to *Caridina typus*, provide an interesting model to understand the possible effects of this history on shaping the biogeography of the IAA and its adjacent ocean basins. Given *C*. *typus*’ planktonic larvae and the consequent ability to disperse across oceans, two alternative scenarios can be predicted regarding this species’ genetic diversity: the first is a widespread distribution with shallow geographic structure as dispersive ability overcomes the geological history of its range; the second is a species complex, where deeper geographic structuring reveals cryptic lineages with restricted distributions. The objective of this study is thus to investigate *C*. *typus* species boundaries, phylogeography and the geographic distribution thereof, using multilocus molecular markers sampled from across the *C*. *typus*’ broad distribution (Fig. 1).

## Results

### DNA – extraction, amplification and sequencing

While the mtDNA markers were fairly easy to amplify and sequence, the nuclear markers (with the exception of anonymous nuclear marker (ANM) Ct33) were very difficult to amplify. We speculate that the missing data for ANM Ct51 might have been caused by the absence of a primer-binding site, since the samples that were not sequenced were either from the same location (Sarawak), or belonged to the same clade in the phylogenetic reconstructions. The partial ribosomal sequence for 28S had the largest amount of missing data due to inability to amplify the locus for many samples. The transportation of the bulk of the samples from the UK to Brazil was delayed by customs issues, and may have led to sample degradation.

### Phylogenetic analyses

**Figure 2:**
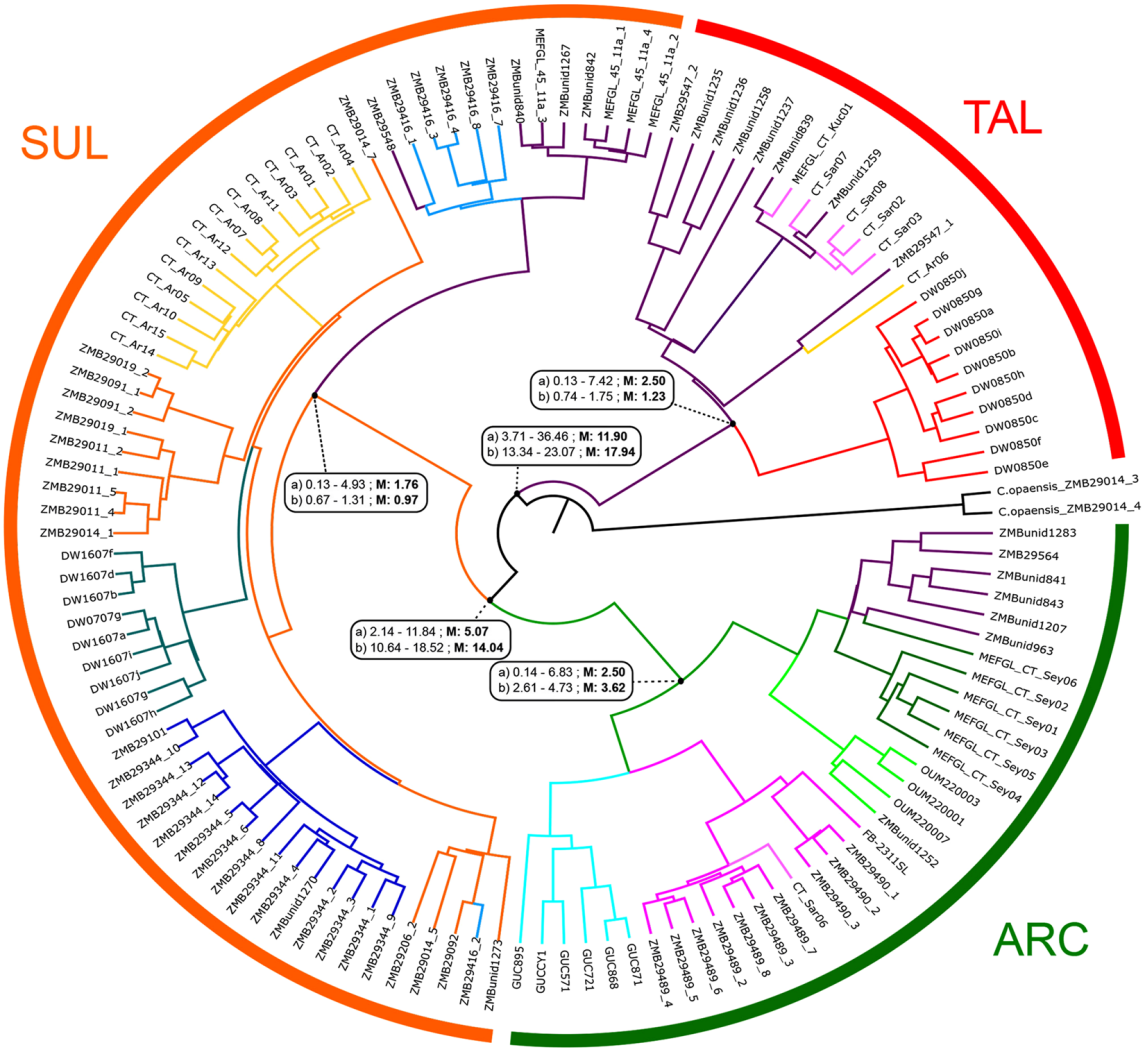
Time-calibrated phylogenetic and phylogeographic relationships in *C*. *typus* rooted using *C*. *opaensis*. Dates are represented as millions of years before present. The main nodes, i.e., those that represent divergence between the major clades and origins of the diversification within them, are labelled with 95% confidence intervals for their dates as well as its median (M). Each label carries the date obtained through data from all markers (**a**) as well as the one obtained solely through mtDNA data (**b**).

The analyses of multiple *Caridina* species revealed *C*. *typus* to be a paraphyletic group (Supplementary Fig. S2), consisting of three morphologically similar clades of ‘*C*. *typus*’ and the morphologically distinct *C*. *villadolidi*. Since morphological data suggest *C*. *villadolidi* is a separate species, and as it was only sequenced for 16S (see Supplementary Table S1), we focussed our analyses on clades of *C*. *typus* morphotypes. We therefore constrained the monophyly of *C*. *typus* to these three clades and excluded *C*. *villadolidi*, which also improved chain convergence. Figure 2 shows the final phylogenetic tree obtained for this data set. Both mitochondrial DNA (mtDNA) and multilocus datasets recovered the same three major clades with some minor differences in the placement of a few individuals: ARC (samples that comprise more or less an arc that surrounds the IAA; see Fig. 1), SUL and TAL. The dates obtained from the multilocus dataset were much more recent compared to those obtained solely from mtDNA (both are presented in Fig. 2). Phylogenetic trees from mtDNA data exhibited longer internal branches at the origin of each clade, with shallow differentiation within the clade. Internal branch lengths were much shorter for multilocus trees.

**Figure 3:**
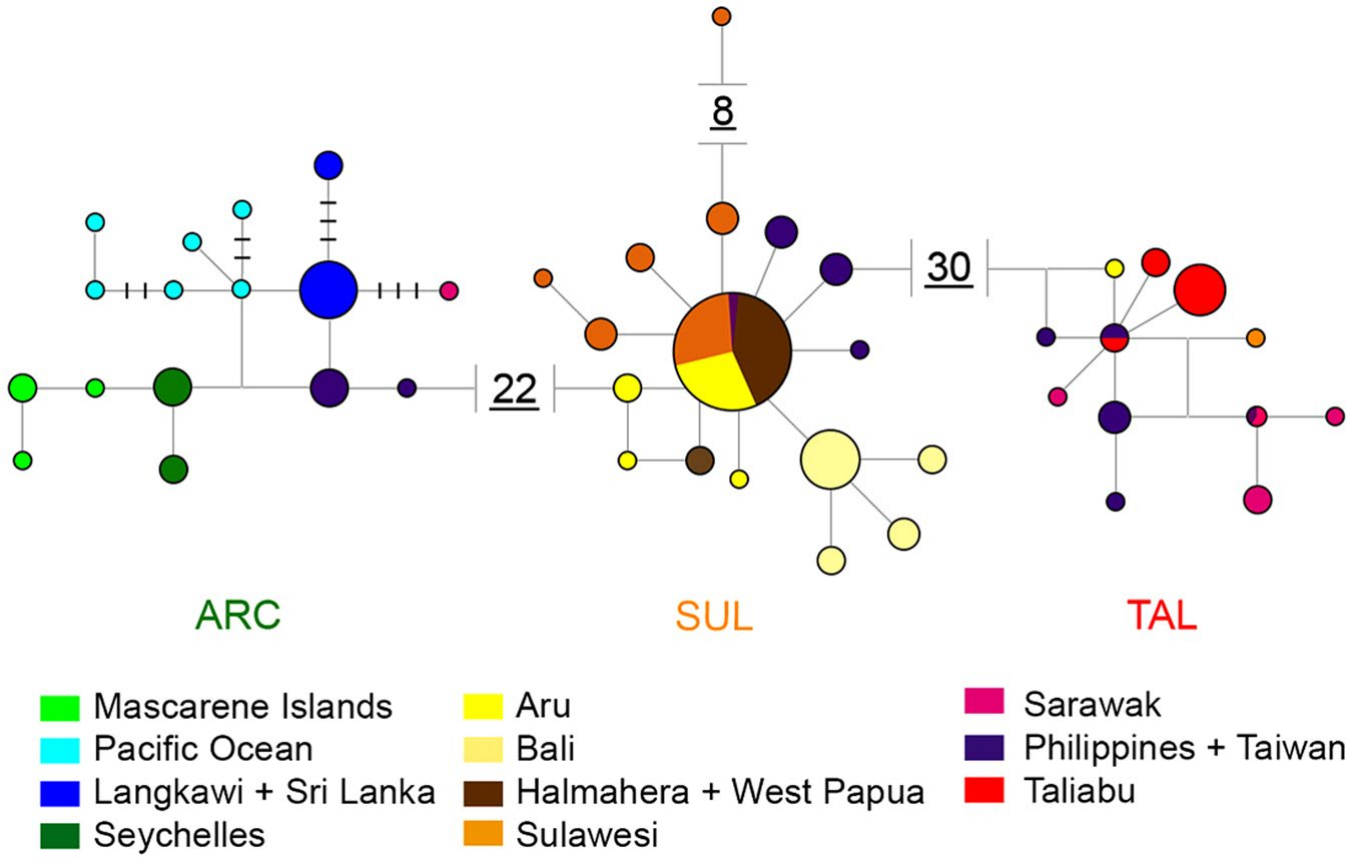
Mitochondrial 16S haplotype network. A single substitution step is represented by a single line connecting two haplotypes. Any number of steps between two and five is represented by the number of dashes on the line connecting two haplotypes. Any distance higher than five steps is represented by the written number of steps. Non-sampled intermediary haplotypes are represented by empty vertices connecting three or more haplotypes. Each colour represents a region according to the legend.

Figure 3 shows the haplotype network for mitochondrial 16S. Haplotype networks for all five markers are presented in Supplementary Fig. S3. The ANM Ct51 (Supplementary Fig. S3d) dataset did not include samples from the ARC clade. The mtDNA networks showed high distinction between the three clades even though they had different internal structures; as expected, cytochrome c oxidase (COI) (Supplementary Fig. S3b) displayed a considerable degree of population structure while 16S (Fig. 3) was more conservative and exhibited little structure in SUL. However, 16S showed some structure in ARC, congruent with the high differentiation found in COI. The ANMs Ct33 and Ct51 showed the most conserved patterns, exhibiting star-like networks and no structure within the clades (Supplementary Fig. S3c,d).

### Species delimitation analyses

All the different species delimitation approaches, either single or multilocus, achieved the same result, with the three main clades identified as different species. Even the Generalised Mixed Yule Coalescent (GMYC) that was conducted with a haplotype tree, and is known to be prone to overestimation of the number of species due to difficulty of the model to assess population structure versus species boundaries^23, 24^, recovered these three distinct clades in all trials with 16S and total mtDNA data (95% confidence interval = 3–4). Trials with only COI had TAL, SUL and ARC divided in two, five and four species respectively (total species number = 11; 95% confidence interval = 4–13). These trials with COI had much higher values for the likelihood ratio test (*LRT* = 1.96 × 10^−4^) than the ones that involved 16S (*LRT* = 6394 × 10^−9^) and 16S + COI (*LRT* = 5.21 × 10^−10^).

BPP showed differences in the results for its discovery and validation approaches. In the validation approach, a very high probability for the existence of three different species was found (*posterior probability* (*pp*) = 0.9722) with a high support for every node (*pp* = 0.9723 for the separation of TAL and 0.9722 for the divergence between ARC and SUL). However, even though the same number of species was obtained by the discovery approach of BPP, the probability was much lower (*pp* = 0.68601), a value that corresponds to the probability of ARC and SUL being different species. The reduced value was due to the relatively high probability of ARC and SUL belonging to the same species (*pp* = 0.30195) compared to the probability of TAL being a separate species (*pp* = 0.98796).

The first trials with STACEY showed a noticeable trend toward more speciose results (10–17 species) but they were far from convergence with very low ESS values. We chose to use very conservative priors and this also appears to have promoted convergence. The final result revealed three main clusters besides the outgroup (see Supplementary Fig. S4).

### Demographic and phylogeographic analyses

Geneland analyses were somewhat problematic due to secondary sympatry – especially in the Philippines – and due to the nuclear markers. When run as a unique set, very different samples from the same location were placed in the same population and no convergence was attained. The SUL clade was particularly troublesome due to the number of common haplotypes across the IAA islands. Running each clade separately, 12 populations were found across the three clades: SUL: Sulawesi, Bali, West Papua and Halmahera, Aru and Philippines_SUL; ARC: Africa (Seychelles and Mascarene Islands), Langkawi (including Sri Lanka), Pacific Islands and Philippines_ARC (including Taiwan); and TAL: Taliabu, Sarawak, and Philippines_TAL. Results for the analysis of molecular variance (AMOVA^25^) are described in Table 1. All three clades exhibited consistent signs of geographic structure in their populations, even though there were differences in the partitioning of genetic diversity between them: whereas ARC and TAL had greater variation structured among populations, the SUL clade showed higher variation structured within populations. All results described by AMOVA were highly significant.

**Table 1:** Results of a two-level AMOVA of genetic differences for both mtDNA and nDNA sequences of the three main clades of the *C*. *typus* species-complex.

Group	mtDNA	nDNA*		p-value		
	Among populations	Within populations	Among populations	Within populations	mtDNA	nDNA*
ARC	66.51	33.49	88.96	11.04	p < 0.00001	0.00196
SUL	20.71	79.29	6.44	93.56	0.00391	p < 0.00001
TAL	99.37	0.63	49.70	50.30	p < 0.00001	p < 0.00001
Total set	92.02	7.98	63.13	36.87	p < 0.00001	p < 0.00001

Notes: Entries represent the percentage of the total variance that is explained by variation within and among the populations defined by Geneland. *Due to the great amount of missing data, the AMOVA for all the samples with nuclear markers was performed with only Ct33, which has the most complete dataset.

Neutrality tests (Table 2) showed conflicting results between Tajima’s *D* and Fu’s *F*_*S*_. Tajima’s *D* values were significantly negative and indicated departures from neutrality only for Sulawesi, but only the Philippine_TAL and Pacific Islands populations showed significant values for neutrality departure for Fu’s *F*_*S*_ under a significance level of *p* < 0.02^26^. Samples from Bali show clear signs of recent expansion in the haplotype networks for both mitochondrial markers (Fig. 3 and Supplementary Fig. S3b), but neither the Sulawesi nor the Pacific Islands populations exhibited the same pattern.

**Table 2:** Results of neutrality tests by population.

Group	Population	Tajima’s D (p-value)	Fu’s Fs (p-value)
ARC	Africa (Seychelles-Mascarene)	0.3845 (0.6770)	–0.7059 (0.2530)
	Langkawi	– 0.9762 (0.1510)	2.0959 (0.8700)
	Pacific Islands	–0.1247 (0.4780)	–3.2830 (**0**.**0080**)*
	Philippines_ARC	–1.1320 (0.1630)	–1.4544 (0.0560)
SUL	Aru	–0.9573 (0.2260)	–1.4558 (0.1220)
	Bali	–0.2726 (0.4030)	–0.7233 (0.2040)
	Halmahera-West Papua	–1.3165 (0.0930)	–0.9136 (0.2260)
	Philippines_SUL	–0.5847 (0.7390)	–0.6744 (0.1990)
	Sulawesi	–1.8258 (**0**.**0110**)	–0.0428 (0.5260)
TAL	Philippines_TAL	–0.5607 (0.3200)	–2.5803 (**0**.**0200**)*
	Sarawak	–0.9726 (0.0980)	–0.8292 (0.0970)
	Taliabu	–0.0203 (0.4920)	–0.3534 (0.3460)

Notes: Values in bold represent results where *p* < 0.05. Values marked with * represent Fu’s *Fs* results where *p* < 0.02.

**Figure 4:**
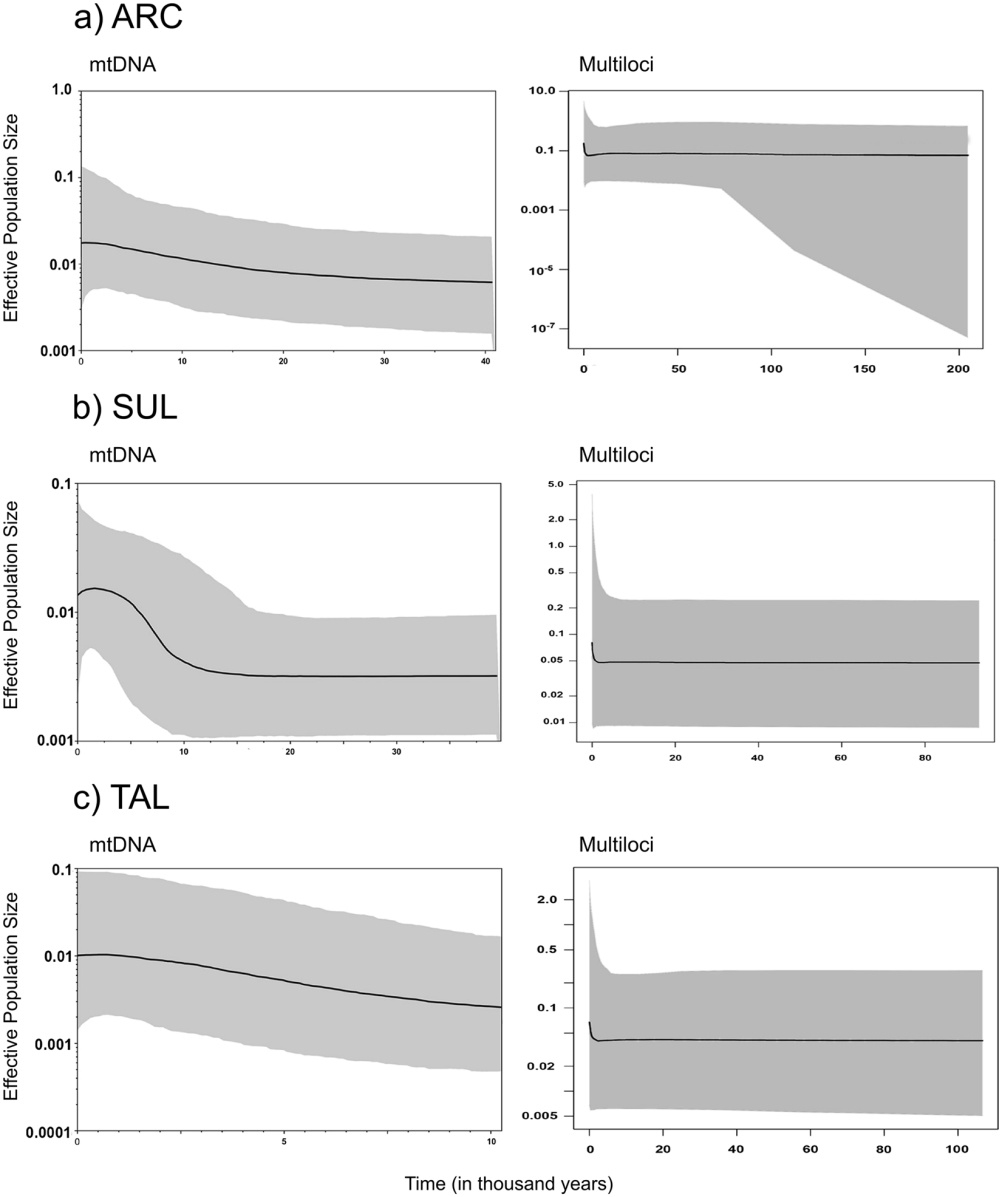
Extended Bayesian skyline plot for mtDNA (left) and multiloci (right) data for each main clade of *C*. *typus*: ARC (**a**), SUL (**b**) and TAL (**c**). The bold line indicates the median whereas the grey area represents the 95% confidence interval. The dates were calibrated with COI substitution rate and are shown in thousands of years before present.

Figure 4 shows the Extended Bayesian skyline plots (EBSPs^27^) for both mtDNA and multilocus datasets. Both ARC and TAL clades show a slight and continuous recent growth for mtDNA (Figs 4a and 3c), whereas SUL shows an abrupt recent expansion (Fig. 4b). When all data is included, all three clades seem to be stable over the last 100 000 years (at least). However, the 95% confidence interval for ARC suggests that either our markers or our sampling was not able to capture a strong expansion trend older than 100 000 years.

BayArea analyses (Supplementary Fig. S5) showed high uncertainty for deeper nodes, while the geographic placement of *C*. *typus*’ ancestor was likely caused by the fact that the outgroup was from Sulawesi and all three major clades were present in the Philippines. However, the more recent nodes show more enlightening patterns. First, the node for the ancestor of TAL (node 248 in Supplementary Fig. S5) has Philippines as the most probable range – and it has a high posterior probability considering that all proposed ranges include the Philippines (Philippines-Taliabu: *pp* = 0.527; Philippines-Taliabu-Sulawesi: *pp* = 0.0879; Philippines: *pp* = 0.0852). Taliabu also shows high probability of being part of the ancestral range. Similarly, Africa is the most probable ancestral range for ARC’s ancestor (node 159 in Supplementary Fig. S5). While most of the possible ranges have two ancestral areas (Africa: *pp* = 0.1944; Africa-Philippines: *pp* = 0.1278; Africa-Pacific: *pp* = 0.1092; Africa-Langkawi and Africa-Sulawesi: *pp* = 0.0639), Africa consistently appears as part of the ancestral range in different combinations. Finally, node 224 (SUL’s ancestor) shows Halmahera as a consistent part of the proposed ancestral ranges, but Sulawesi also has high probability of comprising the ancestral range (Halmahera: *pp* = 0.0905; Halmahera-Sulawesi: *pp* = 0.6152; Halmahera-Philippines-Sulawesi: *pp* = 0.1025). All the probabilities, shown as pie charts, are displayed in Supplementary Fig. S5.

The results of BEAST’s Bayesian discrete phylogeography were inconclusive. The discrete phylogeographic analyses was highly affected by sympatry: the most probable ancestral range of a node was the one most shared across its branches. Moreover, the ancestral range of a specific external branch presented areas occupied by a completely different clade: for example, the node for the samples from the Pacific included Taliabu as a possible part of the ancestral range, even though all Taliabu samples pertained to TAL. That might be because the tree’s dates are too ancient for the method to find a “measurable footprint”^28^. The results are shown in Supplementary Fig. S6.

## Discussion

Our analyses revealed three major clades that cannot be readily explained by the IAA’s geological history alone. As expected for such a vagile species, even though each clade exhibits a fair degree of population structure, the populations are not restricted to a single landmass, and often comprise samples from several islands (Figs 1 and 3). AMOVA tests (Table 1) show that the clade SUL, which comprises most of the IAA, exhibits greater differentiation within populations than between populations, which is well illustrated by the haplotype network recovered for COI (Supplementary Fig. S3b).

This is in agreement with the findings of Fujita *et al*.^29^, whose conclusions were based on less conserved markers and revealed a mixture of two distinct lineages across Japanese islands. Even though the COI region they sequenced overlaps ours by only 167 bp, both of their lineages are placed inside ARC, as we would expect given the similar placement of our Okinawa specimen (see Supplementary Fig. S7).

Thus, based on our data, the three main clades, ARC, SUL and TAL represent independent lineages each with its own history of diversification, expansion and differentiation. Results therefore reflect, at different scales, elements of both of our predictions: a species complex, where a deeper geographic structure reflects restricted distributions, while at a finer scale each of these lineages display shallow phylogeographic structure, suggesting dispersive ability overcomes geological history within each lineage’s range.

**Figure 5:**
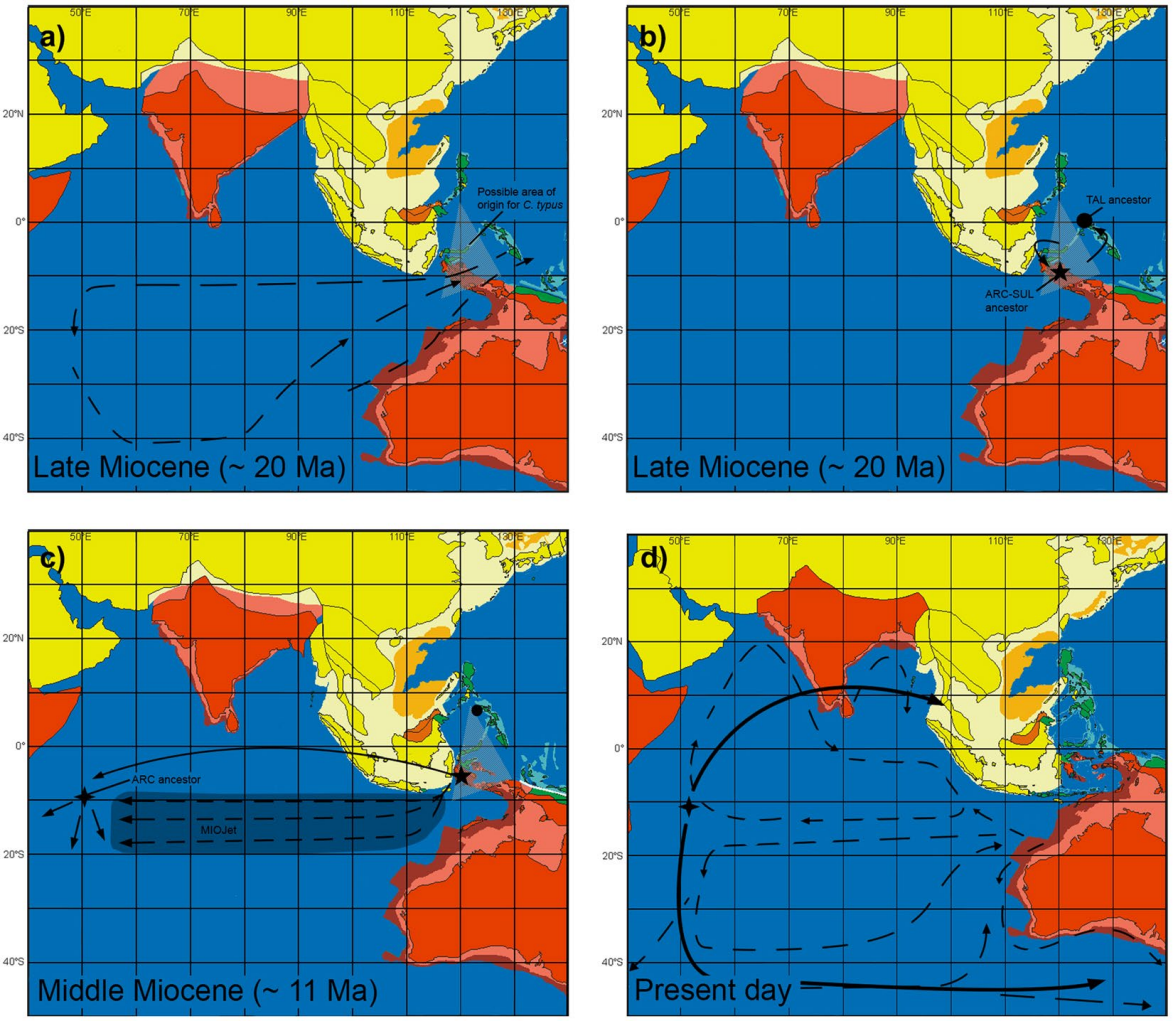
An illustrated suggestion for the biogeographic history of *C*. *typus*. In (**a**), the grey dashed triangle represents the possible ancestral location for the population that would originate based on TAL’s ancestral range inference. The ancestral population would have spread to other locations in the precursor IAA (**b**) probably westwards, to Taliabu, the precursor islands of Sulawesi and/or, based on ARC’s ancestral range inference, the Philippines. With the establishment of the MIOJet, individuals from this ARC-SUL ancestor would have spread to the Indian Ocean and initiated the population that would originate ARC (**c**). The closure of the MIOJet would finally establish currents as they are today and permit the colonisation of Langkawi and the Pacific by ARC. The dashed arrows represent surface currents based on Gourlan *et al*. (2007) and bold arrows represent postulated dispersal events. The dots represent possible points of establishment and isolation of TAL; the star indicates possible points of origin of the ARC-SUL clade; the compass rose indicates the most probable point of origin for the ARC clade. Coastal shelf areas are marked in a lighter tone than the continental areas. Paleomaps were modified from Hall (2012).

TAL shows a high degree of divergence to the other clades, being distant enough to be consistently recovered as a separate clade in the nDNA networks (Supplementary Fig. S3). Moreover, TAL may not be reciprocally monophyletic respective to the other two clades, as it appears to be more closely related to *Caridina villadolidi* (but note, weakly supported in Supplementary Fig. S2), which would make *C*. *typus* a paraphyletic taxon. The EBSP for TAL (Fig. 4) shows a more or less stable population. The slight growth trend recovered by mtDNA could represent a recent expansion in the Philippines, as these samples showed a significant negative value for Fu’s *F*_*S*_. BayArea indicated an ancestral range centred in the Philippines for TAL, with the addition of Taliabu. These results may be an artefact of sampling since these methods can only assess the ancestral ranges if they are included in the current range and sampled – and, in this case, these areas are also time dependent as the Philippine islands were far south of their current position as recently as 5 Ma^18, 30^. Alternatively, as there is clearly a north-south structure (Fig. 2), one possibility would be a Cenozoic range located to the south or east of the Philippines current position (Fig. 5a), a scenario that agrees with Renema *et al*.’s “Miocene Australasian hotspot of biodiversity”^31^.

The lower divergence between ARC and SUL for the nuclear markers may be due to limited sampling for the ARC clade: samples from continental Africa or Asia were not available for analysis, nor have we sampled exhaustively in the Pacific, and both regions could host genetically distinct ARC populations. It is important to note that no ARC samples would amplify at Ct51, which could be a sign of consistent null alleles across the clade and/or mutations in priming sites, and thus it could be speculated that the degree of differentiation between ARC and SUL for that marker should be equal to or higher than the pattern seen in 28S (Supplementary Fig. S3e). Even though the nuclear markers recovered ARC and SUL as different clades (Fig. 2), node dates differed from those recovered solely from mitochondrial data. While mitochondrial data suggest an ARC-SUL divergence around 14 Ma, multiloci analyses indicated a much more recent cladogenesis at ~5 Ma. It must be stressed that molecular dating is very imprecise with very large confidence intervals, and is presented to give a general idea of the geological age of divergences and to provide discussion points for biogeographic hypotheses. These dates, in spite of their difference, however, provide a working hypothesis associated with the MIOJet. Once the MIOJet was established, around 14 Ma according to Gourlan *et al*.^22^, individuals from an ancestral population (most likely SUL or TAL) could have dispersed westwards (Fig. 5c) and colonised the Seychelles, and later the Mascarene Islands, whose oldest island, Mauritius, began to emerge around 8 Ma^16^. At ~3.5 Ma, with the closure of the Indonesian Gateway and the termination of the MIOJet, contact between ARC and SUL would have been terminated.

With the closure of the MIOJet, ARC individuals may have become able to move eastwards and to colonise Malaysia and the Pacific (Fig. 5d). Results from BayArea analyses support that scenario, with Africa a likely part of ARC’s ancestral range. The Philippines is interesting in this scenario: BayArea shows a considerable probability of the Philippines being part of ARC’s ancestral range, yet in spite of the amount of missing data, a clear separation was evident between ARC samples from the Indian and Pacific Oceans (Fig. 2). The Philippines could indeed have been part of the ancestral range as the starting point for ARC’s MIOJet-mediated dispersal (Fig. 5b), but if this were the case, ARC-SUL divergence would have occurred before ARC’s dispersal throughout the Indian Ocean.

EBSPs for mitochondrial data (Fig. 4) depict an abrupt expansion for SUL and, although not statistically significant, a slight steady growth trend for both ARC and TAL, whilst the plots obtained for all markers show signs of growth only for ARC. This is congruent with the MIOJet scenario, where ARC should have had a somewhat constant spatial expansion for the past 14 Ma, and more so over the last ~3.5 Ma. Fu’s *F*_*S*_ shows a significant sign of recent expansion for the samples from the Pacific Islands (Australia, Japan, Vanuatu, New Caledonia; Table 2), which is also in agreement with the MIOJet scenario, but could be artefactual due to limited sampling for that region. The same significance is not found for Tajima’s *D* value, but *F*_*S*_ should be more sensitive to departures from neutrality^26^.

The mtDNA EBSP for SUL (Fig. 4) exhibits a signature of a recent expansion that agrees with patterns found using AMOVA and haplotype networks. Nonetheless, the lack of structure and high gene flow between islands may be more in agreement with constant demographic size depicted by the total evidence from all five markers. Departure from neutrality in Tajima’s *D* was only significant for the Sulawesi population, but it does not show significant *F*_*S*_ values. These results could represent the colonisation of several Indonesian islands following the end of the MIOJet, since the dates found for the origin of SUL are very recent, but could also be related to female philopatry.

The question raised by these patterns and findings is, are there any ecological or developmental differences between each clade? Atyids’ life history could be associated with their egg size^4, 7^, and in this context, *C*. *typus*’ hardy planktonic larvae can be related to its dispersal ability. However, while ARC is exceptionally widespread across both the eastern and western hemispheres, SUL and TAL are restricted to the IAA, which could raise questions on the differences between each clade’s eggs and larvae. Differences of that kind have been shown in other studies on *Caridina* ^8^, even though differences in vagility were not as obvious in that case. Fujita *et al*.^29^ showed that Japanese *C*. *typus* has a considerable dispersal ability, but, as far as we can tell, their samples are restricted to our ARC clade. In the early Miocene, when the Indonesian Gateway was wide open, eggs and larvae could have been carried toward the rest of the IAA from *C*. *typus*’ original range (Fig. 5a)^18^ and established the ancestral ARC and SUL populations (Fig. 5b), probably in the southerly part of those ranges (given the BayArea results), with Halmahera and Sulawesi the most likely ancestral ranges. The dates for TAL’s divergence from ARC-SUL range from ~18 Ma (mtDNA) to ~12 Ma (based on the multilocus dataset), which may indicate that gene flow between TAL and the ARC-SUL’s ancestral population became restricted by the MIOJet and by the formation of Sulawesi and other islands of the IAA, as the SE Asian Gateway narrowed significantly by 5 Ma. If the same dispersive ability was present in all three clades, ecological exclusion could explain why ARC was not able to recolonise the IAA (except for the Philippines), and why SUL and TAL’s individuals did not also establish populations of their own outside the IAA.

Sukumaran and Knowles^32^ have pointed out that coalescent-based methods of species delimitation may not track speciation, but rather tracks emergent lineages that represent population structure, thus overinflating the number of species identified. Even though their simulations were run with BPP – which was a method used in this paper – and albeit we agree that species delimitation should be approached with caution, two (GMYC and STACEY) of the three methods used here were based on mixed models. Due to the fact that no morphological differences between the three clades described here have been identified to date (pers. comms. Werner Klotz & Sammy De Grave), we were very conservative in the analysis and favoured a single species where possible. Methods such as GMYC and BPP depend solely on the input tree and sequences, but STACEY depends on convergence, and thus on priors. While setting informative empirical priors, we conducted several trials favouring different numbers of species, but we aimed to design a robust test for the null hypothesis that *C*. *typus* comprises a single monophyletic species. The three *C*. *typus* clades identified in our analyses represent very distinct lineages and the fact that all species delimitation approaches, even those that have been shown to be prone to overestimation like GMYC, agreed on the final result strongly suggests the samples included here represent three distinct species. We do, however, acknowledge the risk of a false positive^32^ from the methods used here, and highlight the need for future morphological and ecological studies to test these results.

Many levels of sympatry were found: two individuals from Aru and from Sarawak were consistently recovered as belonging to TAL and ARC respectively, and individuals from the Philippines were roughly equally divided between the three clades. This study did not find any conclusive evidence for hybridization between clades, but the majority of the Philippines’ samples were sequenced only for mitochondrial 16S, which can not reveal hybridisation. Efforts toward a more detailed morphological description of the specimens could reveal for the Philippines, for instance, a similar scenario to that of von Rintelen *et al*.’s study on Sulawesi lakes^33^, that found similar populations diverging along the same habitat, which in this case could occur following secondary contact. Alternatively, we may find a late stage in radiation^34^ like that found for *C*. *ensifera* ^9^, with prezygotic isolating mechanisms playing a role. Finally, we agree with Page *et al*.’s suggestion that egg and larva size assessment between clades should be conducted, and may inform on differences in vagility, and thus perhaps evolutionary patterns, among the three clades^8^.

## Conclusion

This study has shown that *C*. *typus*, as currently defined under morphological criteria, comprises three distinct clades with unique evolutionary histories. One clade, ARC, may be a useful model taxon for future research due to its extremely wide distribution and clear separation between populations from the Indian Ocean and those from the Pacific Ocean. The MIOJet may have played an important role in this clade’s history. Dispersal *via* the MIOJet has been associated with the evolution of several taxa^35, 36^ and our data and results fit this hypothesis well. Moreover, samples from the IAA, mostly pertaining to SUL and TAL, raise some interesting questions about *C*. *typus*’ evolution, such as is there sympatry in Sarawak (ARC-TAL) or Aru (SUL-TAL), or is sympatry restricted to the Philippines? Do SUL and TAL exclude each other ecologically across the IAA? Is there philopatry in *C*. *typus* populations? To address these issues, further sampling is necessary, especially in the Philippines and the Pacific islands. Further marker sampling will also allow to test introgression between clades, which still cannot be ruled out.

Another important result of our study are questions raised about the monophyly and taxonomic status of *C*. *typus*. We conservatively identified three distinct clades (which could potentially be more since many locations were not sampled) and found evidence to treat each clade as a unique species. In addition, preliminary results indicate that these three clades may not be monophyletic, as TAL could be more closely related to *C*. *villadolidi* (though, as discussed, weakly supported; see Supplementary Fig. S2). Thus, *Caridina typus*’ taxonomy is not clear, for instance, no type-locality was originally described, although it appears to be Mauritius^3^. This indicates that a full taxonomic revision is required.

## Materials and Methods

### Sampling

We sampled 117 *C*. *typus* specimens whose IDs and locations are listed in Supplementary Table S1 and plotted on the map in Fig. 1. We sampled populations throughout the Indo-Australian Archipelago, and from other locations in *C*. *typus*’ range, including Taiwan, the Seychelles, and Mascarene Islands. The collections were conducted between 2003 and 2016, and samples were preserved in 100% ethanol. The samples are deposited in the Museum für Naturkunde Berlin (ZMB), Museum Victoria (CT_ArXX), Oxford University Museum of Natural History (OUMNH) and in the Molecular Ecology and Fisheries Genetics Laboratory at Bangor University. In our alignment, we added seven mitochondrial sequences from GenBank (see Supplementary Table S1) that expanded our sampling to Australia, Japan, New Caledonia, Vanuatu (these localities are referred to as Pacific Islands in the main text) and Sri Lanka. In this study, we also included five samples of *C*. *villadolidi* Blanco, 1939, which has historically been a synonym of *C*. *typus* var. *longirostris* De Man, 1892^37, 38^ and two specimens of *C*. *opaensis* J. Roux, 1904, here used as outgroup.

### DNA – extraction, amplification and sequencing

To extract DNA, Promega’s Wizard® Genomic DNA Purification Kit was used on a pair of pleopods following product instructions. We then amplified two mitochondrial loci (a 517 bp fragment of 16S and a 783 bp fragment of COI), and three nuclear loci (a 374 bp ANM named Ct33, a 330 bp ANM named Ct51, and a 368 bp fragment of the D2 segment of 28S). PCR reactions were conducted including the following reagents: 1–2 μL template DNA, 2.5 μL reaction buffer (1X), 1.25 μL MgCl_2_ (50 mM), 2.5 μL dNTP’s, 2.0 μL of each primer, 0.24 μL of 5.0 U/μL Platinum Taq Polymerase (Thermo Fischer) and sterilised distilled water to mix 25 μL. Only 16S and 28S had 1.0 μL MgCl_2_. The standardised program and the primers used to amplify each marker are shown in Table 3. PCR products were purified with Beckman Coulter’s Agencourt AMPure® system except for Ct33, which was purified with Affymetrix’s ExoSAP-IT® kit since its PCR products were frequently lost in purification through the AMPure system. The same primers used in amplification were used for sequencing. Sequencing was performed on an ABI 3130 sequencer using ABI Big Dye terminator chemistry (Applied Biosystems). All sequences were deposited in GenBank (accession numbers KY069328–KY069787 and KY436221–KY436224).

**Table 3:** Loci, primer sequences and PCR conditions utilised for *C*. *typus*.

Marker	Primer	Sequence	Program
16S	16S-F-Car	5′-TGC CTG TTT ATC AAA AAC ATG TC-3′^9^	95 °C – 2 min; 40x [95 °C – 30 s, 50 °C – 30 s, 72 °C – 30 s]; 72 °C – 10 min
	16S-R-Car	5′-AGA TAG AAA CCA ACC TGG CTC-3′^9^	
COI	COI-F-Car	5′-GCT GCT AAT TTT ATA TCT ACA G-3′^9^	95 °C – 2 min; 40x [95 °C – 30 s, 45 °C – 30 s, 72 °C – 30 s]; 72 °C – 10 min
	COI-R-Car	5′-TGT GTA GGC ATC TGG GTA ATC-3′^9^	
Ct33	CT33-F	5′-CCT TTC TAG ACG CAT CAA TGG-3′	95 °C – 2 min; 10x [95 °C – 35 s, 63 °C – 35 s (-0.5 °C/cycle), 72 °C – 1 min]; 10x [95 °C – 35 s, 58 °C – 35 s, 72 °C – 1 min]; 15x [95 °C – 35 s, 52 °C – 35 s, 72 °C – 1 min] 72 °C – 10 min^69^
	CT33-R	5′-ATC TGA TTG GCT GGC TGA AT-3′	
Ct51	CT51-F	5′-GGG CTT TTA GCT AAG CTC TCG-3′	
	CT51-R	5′-GGC AGT TTC TTA TGG GCA TT-3′	
28 S	28SD3AP	5′-CAA GTA CCG TGA GGG AAA GTT G-3′^70^	95 °C – 4 min; 10 × [95 °C – 30 s, 50 °C – 45 s, 72 °C – 2:30 min]; 18 × [94 °C – 30 s, 45 °C – 45 s, 72 °C – 2:30 min]; 72 °C – 10 min
	D3-4283 R	5′-TAG TTC ACC ATC TTT CGG GTC-3′^71^	

### Phylogenetic analyses

In order to assess *C*. *typus*’ monophyly, multiple *Caridina* species’ 16S sequences taken from GenBank were used in different combinations with our dataset. This analysis aimed only to give us an informative prior about the relationships within *C*. *typus* due to the inclusion of *C*. *villadolidi*, since our first limited trials depicted it as either a paraphyletic or a polyphyletic group. There was considerable difficulty amplifying all loci for some of the samples, thus we constructed several different datasets and many trials in order to obtain informative empirical priors, and to assess and minimise the effects of missing data. From those initial trials, two datasets were retained for subsequent analyses: one comprising only mtDNA data, and a second dataset including all five alignments.

The ANMs and COI were aligned with ClustalW^39, 40^ as implemented in MEGA6^41^. The mitochondrial ribosomal DNA fragment, 16S, was aligned through MAFFT^42^, while the nuclear ribosomal DNA fragment, 28S, was manually aligned through secondary structure (see Supplementary Fig. S1). The different alignment methods were due to the nature of the markers: autosomal and protein-coding markers were straightforward to align and homologous positions were easily identified by the position of nucleotides or codons. Ribosomal markers’ homology is intertwined with the secondary ribosomal structure and more information from the marker can be accessed this way. Our 16S sequences were perhaps too short to exhibit the need for a manual alignment, but the presence of several gaps was problematic to ClustalW, thus we used MAFFT. Network 4.6^43^ was then used to build a median-joining network^44^ for each marker, taking gaps into consideration for all loci except COI that had sequences of different lengths cut down to the same size.

The best substitution model was chosen through the corrected Akaike Information Criterion (AICc) implemented in jModelTest 2.1^45^: HGY + Γ for 16S, GTR + I + Γ for COI, JC for Ct33, HKY + Γ for Ct51 and GTR for 28S. In order to choose the best molecular clock model, we ran multiple analyses for each marker separately with either a strict or relaxed molecular clock (both exponential and log normal models were tested), and we then used TRACER v1.6^46^ to compare log values using an AICM approach as described by Baele *et al*.^47^.

### Priors and model setting

For the monophyly assessment trials, we ran a coalescent clock based analysis in standard BEAST 2 v. 2.3.0^48^ using a reversible-jump method to choose the best substitution model^49, 50^. In addition to the clock based analysis, we also conducted a non-clock analysis with MrBayes 3.2.5^51^ to assess the rooting recovered by BEAST2. The input file was run for 10 000 000 generations, with four simultaneous chains, and sampling every 1 000 generations.

For both mtDNA and multilocus datasets, we also ran a coalescent clock based analysis for these samples, but this time we used *BEAST^48^ in order to use multiple alignments. A reversible-jump method was used to choose the best substitution model and the runs were visually diagnosed through TRACER. The molecular clock models used for each locus were selected through AICM analysis: relaxed lognormal clock for the mitochondrial markers and strict clock for the nuclear markers. A constraint in the ingroup monophyly (*C*. *typus* specimens) was also used based on the results of the monophyly assessment and only proper prior distributions were chosen, with the exception of the clock rate prior for strict clocks, which were set to a proper bounded uniform distribution. Each analysis was run for 500 000 000 generations and sampled every 10 000 generations. Runs were repeated 3–5 times to assess the repeatability of the results, and a 50% burn-in was used in *TreeAnnotator*.

A substitution rate of 1.1–1.3% per million years for COI was used to date the cladogenesis events. This rate was taken from the divergence rates presented by Knowlton *et al*.^52^ for a caridean shrimp based on the rise of the Isthmus of Panama and it was previously used for a *Caridina* species by Hurwood & Hughes^53^. Since it is somewhat close to the COI rate identified by Brower^54^, it should be a conservative approach to estimate node ages. In the preliminary analyses and trials, several inconsistences between the dates of the COI trees and the multilocus trees were found, as well as difficulty in MCMC convergence for the multilocus analyses. In order to avoid age inflation or deflation and to facilitate the convergence of the runs, informative priors on the relative rates of the other loci were estimated in Garli^55^ following the method described in Marshall *et al*.^56^.

The priors and models were tested in several trials with different combinations of priors’ distributions, and markers. All analyses in BEAST, MrBayes, jModelTest and Garli were run in CIPRES^57^.

### Species delimitation analyses

To use the GMYC approach on species delimitation^58, 59^, we needed a mtDNA alignment that included only unique haplotypes. The Bayesian approach of the same method (bGMYC) introduced by Reid & Carstens^60^ was also used. Even though both GMYC and bGMYC are different mathematical implementations of the same model, and the latter is known for reducing uncertainty of the former, we preferred to include both sets of results for our data in order to analyse and identify possible causes for differences between them. For the same reason, trials were made for separate 16S and COI trees to check for congruence between loci.

For the multilocus dataset, we used the package STACEY^61, 62^ implemented in BEAST2 to infer minimal clusters from each dataset. We used very conservative priors and favoured a single cluster (e.g. high values for collapse weight (*ω*) and smaller values for collapse height (ε)) in order to avoid overestimating the number of species. The analysis was repeated twice, and run for one billion generations sampling every 100 000 generations.

Another method used was the discovery method implemented in BPP v.3.2^63, 64^. The results from all the species discovery analyses were used as input to the validation method of BPP. Both BPP approaches had MCMC samples taken every 2 iterations with a total 2 000 000 samples and a 10 000 iteration burn-in plus a *thetaprior* = *2* 2000 and *tauprior* = *2* 1000.

### Demographic and phylogeographic analyses

Geneland^65, 66^ was used as an approach to delimit populations inside each main clade. Haplotype networks were inconclusive and we chose not to apply geographic distance. Due to sympatry, each clade was run separately and due to the amount of missing data present for nuclear markers, only mtDNA data were run. Three independent runs were set with 10 000 000 iterations with a maximum of 20 populations. We then used Arlequin 3.5^67^ to perform an AMOVA for both mitochondrial and nuclear loci. The same software was used to conduct two neutrality tests (Tajima’s *D* and Fu’s *F*_*S*_) in order to identify any signatures of demographic change. Again, neutrality tests were conducted only for 16S due to excessive amounts of missing data for nuclear markers and COI. EBSPs were also used to assess the demographic history of each clade, run in BEAST2 with the same respective parameters for mtDNA and multilocus phylogenetic analyses.

To reconstruct the ancestral range, we used RASP^68^ to apply the BayArea model. This model was chosen due to the feature of implementing distance to the probability of dispersal given that our studied organism is quite widespread. BayArea was run for 5 000 000 generations sampling every 1 000 generations with a TRUE setting for geographic distance power. For reasons of convergence and limitations in the number of locations accepted by BayArea, the populations identified by Geneland were taken into account for each sample’s location assignment. We also used the Bayesian discrete phylogeography method^28^ implemented in BEAST with the same parameters as applied to our phylogenetic analyses. Convergence and number of locations was not a problem with this analysis, but to ensure comparability between methods, we used the same location assignments as in BayArea. The phylogeographic analyses were run only for the multilocus dataset.

### Data availability

The datasets (DNA sequences) generated and analysed during the current study are available in GenBank. Accession numbers for each individual sample are available in Supplementary Table S1.

## Electronic supplementary material

Supplementary Information
