# Supplementary material for: The complex evolutionary history and phylogeography of Caridina typus (Crustacea: Decapoda): long-distance dispersal and cryptic allopatric species
Source: Sci Rep. 2017 Aug 22;7:9044. doi: 10.1038/s41598-017-08494-w (PMC5567278; doi:10.1038/s41598-017-08494-w)
Supplement: Supplementary file 1 — Supplementary Information [file 41598_2017_8494_MOESM1_ESM.pdf]

The complex evolutionary history and phylogeography of *Caridina typus* (Crustacea: Decapoda): long-distance dispersal and cryptic allopatric species

Samuel C. Bernardes, Almir R. Pepato, Thomas von Rintelen, Kristina von Rintelen, Timothy J. Page, Hendrik Freitag, Mark de Bruyn

Table S1: Samples used in this study

Taxon	Location		Sample ID	Coordinates		GenBank accession numbers				
						16S	COI	Ct33	Ct51	28S
Caridina typus	Indonesia	Aru	CT_Ar01	06° 10' 42.53" S	134° 18' 04.30" E	KY069330	KY069449	KY069546	KY069642	
			CT_Ar02	06° 10' 42.53" S	134° 18' 04.30" E	KY069331	KY069450	KY069547	KY069643	KY069719
			CT_Ar03	06° 10' 42.53" S	134° 18' 04.30" E	KY069332	KY069451	KY069548	KY069644	
			CT_Ar04	06° 10' 42.53" S	134° 18' 04.30" E	KY069333	KY069452	KY069549	KY069645	KY069720
			CT_Ar05	06° 10' 42.53" S	134° 18' 04.30" E	KY069334	KY069453	KY069550	KY069646	KY069721
			CT_Ar06	06° 10' 42.53" S	134° 18' 04.30" E	KY069335	KY069454	KY069551	KY069647	KY069722
			CT_Ar07	06° 10' 42.53" S	134° 18' 04.30" E	KY069336	KY069455	KY069552	KY069648	KY069723
			CT_Ar08	06° 10' 42.53" S	134° 18' 04.30" E	KY069337	KY069456	KY069553	KY069649	KY069724
			CT_Ar09	06° 10' 42.53" S	134° 18' 04.30" E	KY069338	KY069457	KY069554	KY069650	KY069725
			CT_Ar10	06° 10' 42.53" S	134° 18' 04.30" E	KY069339	KY069458	KY069555	KY069651	KY069726
			CT_Ar11	06° 10' 42.53" S	134° 18' 04.30" E	KY069340	KY069459	KY069556	KY069652	KY069727
			CT_Ar12	06° 10' 42.53" S	134° 18' 04.30" E	KY069341	KY069460	KY069557	KY069653	
			CT_Ar13	06° 10' 42.53" S	134° 18' 04.30" E	KY069342	KY069461	KY069558	KY069654	
			CT_Ar14	06° 10' 42.53" S	134° 18' 04.30" E	KY069343	KY069462	KY069559	KY069655	
			CT_Ar15	06° 10' 42.53" S	134° 18' 04.30" E	KY069344	KY069463	KY069560	KY069656	
		Bali	ZMB29101	08° 37' 56.70" S	115° 08' 08.58" E	KY069395	KY069514	KY069611	KY069692	
			ZMB29344_1	08° 30' 44.76" S	115° 01' 22.20" E	KY069397	KY069516	KY069613	KY069693	KY069769
			ZMB29344_2	08° 30' 44.76" S	115° 01' 22.20" E	KY069403	KY069522	KY069619	KY069699	
			ZMB29344_3	08° 30' 44.76" S	115° 01' 22.20" E	KY069404	KY069523	KY069620	KY069700	
			ZMB29344_4	08° 30' 44.76" S	115° 01' 22.20" E	KY069405	KY069524	KY069621	KY069701	
			ZMB29344_5	08° 30' 44.76" S	115° 01' 22.20" E	KY069406	KY069525	KY069622	KY069702	KY069773
			ZMB29344_6	08° 30' 44.76" S	115° 01' 22.20" E	KY069407	KY069526	KY069623	KY069703	KY069774
			ZMB29344_8	08° 30' 44.76" S	115° 01' 22.20" E	KY069408	KY069527	KY069624	KY069704	
			ZMB29344_9	08° 30' 44.76" S	115° 01' 22.20" E	KY069409	KY069528	KY069625	KY069705	KY069775
			ZMB29344_10	08° 30' 44.76" S	115° 01' 22.20" E	KY069398	KY069517	KY069614	KY069694	KY069770
			ZMB29344_11	08° 30' 44.76" S	115° 01' 22.20" E	KY069399	KY069518	KY069615	KY069695	KY069771
			ZMB29344_12	08° 30' 44.76" S	115° 01' 22.20" E	KY069400	KY069519	KY069616	KY069696	
			ZMB29344_13	08° 30' 44.76" S	115° 01' 22.20" E	KY069401	KY069520	KY069617	KY069697	
			ZMB29344_14	08° 30' 44.76" S	115° 01' 22.20" E	KY069402	KY069521	KY069618	KY069698	KY069772
			ZMBunid1270**	08° 21' 00.00" S	114° 37' 60.00" E	KY069438				

Halmahera	ZMB29416_1	00° 53' 35.22" N	127° 37' 17.22" E	KY069410	KY069529	KY069626	KY069715/KY069716	KY069776
	ZMB29416_2	00° 53' 35.22" N	127° 37' 17.22" E	KY069411	KY069530	KY069627	KY069706	KY069777
	ZMB29416_3	00° 53' 35.22" N	127° 37' 17.22" E	KY069412	KY069531	KY069628	KY069707	
	ZMB29416_4	00° 53' 35.22" N	127° 37' 17.22" E	KY069413	KY069532	KY069629	KY069708	KY069778
	ZMB29416_7	00° 53' 35.22" N	127° 37' 17.22" E	KY069414	KY069533	KY069630	KY069709	KY069779
	ZMB29416_8	00° 53' 35.22" N	127° 37' 17.22" E	KY069415	KY069534	KY069631	KY069710	
Sulawesi	ZMB29011_1	03° 40' 30.06" S	122° 13' 41.88" E	KY069383	KY069502	KY069599	KY069681	KY069756
	ZMB29011_2	03° 40' 30.06" S	122° 13' 41.88" E	KY069384	KY069503	KY069600	KY069682	KY069757
	ZMB29011_4	03° 40' 30.06" S	122° 13' 41.88" E	KY069385	KY069504	KY069601	KY069683	KY069758
	ZMB29011_5	03° 40' 30.06" S	122° 13' 41.88" E	KY069386	KY069505	KY069602	KY069684	KY069759
	ZMB29014_1	04° 23' 17.04" S	122° 35' 33.18" E	KY069387	KY069506	KY069603	KY069685	KY069760
	ZMB29014_5	04° 23' 17.04" S	122° 35' 33.18" E	KY069388	KY069507	KY069604	KY069686	KY069761
	ZMB29014_7	04° 23' 17.04" S	122° 35' 33.18" E	KY069389	KY069508	KY069605	KY069687	KY069762
	ZMB29019_1	03° 40' 59.70" S	122° 15' 17.88" E	KY069390	KY069509	KY069606	KY069688	KY069763
	ZMB29019_2	03° 40' 59.70" S	122° 15' 17.88" E	KY069391	KY069510	KY069607	KY069689	KY069764
	ZMB29091_1	02° 56' 03.00" S	121° 06' 57.60" E	KY069392	KY069511	KY069608	KY069690	KY069765
	ZMB29091_2	02° 56' 03.00" S	121° 06' 57.60" E	KY069393	KY069512	KY069609	KY069691	KY069766
	ZMB29092	05° 00' 57.60" S	119° 40' 55.20" E	KY069394	KY069513	KY069610	KY069711/KY069712	KY069767
	ZMB29206_2	08° 30' 44.76" S	121° 04' 45.00" E	KY069396	KY069515	KY069612	KY069713/KY069714	KY069768
	ZMBunid1273**	04° 04' 37.80" S	121° 40' 04.20" E	KY069439				
Taliabu	DW0850a	______________	______________	KY069351	KY069470	KY069567	KY069658	KY069732
	DW0850b	______________	______________	KY069352	KY069471	KY069568	KY069659	KY069733
	DW0850c	______________	______________	KY069353	KY069472	KY069569	KY069660	KY069734
	DW0850d	______________	______________	KY069354	KY069473	KY069570	KY069661	KY069735
	DW0850e	______________	______________	KY069355	KY069474	KY069571	KY069662	KY069736
	DW0850f	______________	______________	KY069356	KY069475	KY069572	KY069663	KY069737
	DW0850g	______________	______________	KY069357	KY069476	KY069573	KY069664	KY069738
	DW0850h	______________	______________	KY069358	KY069477	KY069574	KY069665	KY069739
	DW0850i	______________	______________	KY069359	KY069478	KY069575	KY069666	KY069740
	DW0850j	______________	______________	KY069360	KY069479	KY069576	KY069667	KY069741
West Papua	DW1607a	00° 12' 06.10" S	130° 48' 48.60" E	KY069361	KY069480	KY069577	KY069668	KY069742
	DW1607b	00° 12' 06.10" S	130° 48' 48.60" E	KY069362	KY069481	KY069578	KY069669	
	DW1607d	00° 12' 06.10" S	130° 48' 48.60" E	KY069363	KY069482	KY069579	KY069670	KY069743

		DW1607f	00° 12' 06.10" S	130° 48' 48.60" E	KY069364	KY069483	KY069580		KY069671	
		DW1607g	00° 12' 06.10" S	130° 48' 48.60" E	KY069365	KY069484	KY069581		KY069672	KY069744
		DW1607h	00° 12' 06.10" S	130° 48' 48.60" E	KY069366	KY069485	KY069582		KY069673	KY069745
		DW1607i	00° 12' 06.10" S	130° 48' 48.60" E	KY069367	KY069486	KY069583		KY069674	KY069746
		DW1607j	00° 12' 06.10" S	130° 48' 48.60" E	KY069368	KY069487	KY069584		KY069675	KY069747
		DW0707	____________	____________	KY069350	KY069469	KY069566		KY069657	
Malaysia	Langkawi	ZMB29489_2	06° 24' 07.52" N	099° 49' 10.95" E	KY069416	KY069535	KY069632			KY069780
		ZMB29489_3	06° 24' 07.52" N	099° 49' 10.95" E	KY069417	KY069536	KY069633			KY069781
		ZMB29489_4	06° 24' 07.52" N	099° 49' 10.95" E	KY069418	KY069537	KY069634			KY069782
		ZMB29489_5	06° 24' 07.52" N	099° 49' 10.95" E	KY069419	KY069538	KY069635			KY069783
		ZMB29489_6	06° 24' 07.52" N	099° 49' 10.95" E	KY069420	KY069539	KY069636			KY069784
		ZMB29489_7	06° 24' 07.52" N	099° 49' 10.95" E	KY069421	KY069540	KY069637			KY069785
		ZMB29489_8	06° 24' 07.52" N	099° 49' 10.95" E	KY069422	KY069541	KY069638			KY069786
		ZMB29490_1	06° 25' 24.18" N	099° 40' 02.85" E	KY069423	KY069542	KY069639			
		ZMB29490_2	06° 25' 24.18" N	099° 40' 02.85" E	KY069424	KY069543	KY069640			KY069787
		ZMB29490_3	06° 25' 24.18" N	099° 40' 02.85" E	KY069425	KY069544	KY069641			
	Sarawak	CT_Sar02	01° 43' 04.71" N	110° 27' 24.43" E	KY069345	KY069464	KY069561			KY069728
		CT_Sar03	01° 43' 04.71" N	110° 27' 24.43" E	KY069346	KY069465	KY069562			KY069729
		CT_Sar06	01° 43' 04.71" N	110° 27' 24.43" E	KY069347	KY069466	KY069563			
		CT_Sar07	01° 43' 04.71" N	110° 27' 24.43" E	KY069348	KY069467	KY069564			KY069730
		CT_Sar08	01° 43' 04.71" N	110° 27' 24.43" E	KY069349	KY069468	KY069565			KY069731
		MEFGL_CT_Kuc01	01° 41' 20.64" N	109° 51' 10.92" E	KY069373	KY069492	KY069589		KY069680	KY069748
Philippines	Bohol	ZMB29564	09° 38' 40.14" N	124° 09' 35.52" E	KY069429					
	Luzon	ZMBunid1207**	14° 28' 49.20" N	122° 56' 33.00" E	KY069430					
		ZMBunid1235**	14° 47' 50.96" N	120° 19' 03.55" E	KY069431					
		ZMBunid1236**	14° 47' 50.96" N	120° 19' 03.55" E	KY069432					
		ZMBunid1237**	14° 47' 50.96" N	120° 19' 03.55" E	KY069433					
		MEFGL_45_11a_1	14° 47' 18.84" N	120° 16' 12.12" E	KY069369	KY069488	KY069585		KY069676	
		MEFGL_45_11a_2	14° 47' 18.84" N	120° 16' 12.12" E	KY069370	KY069489	KY069586		KY069677	
		MEFGL_45_11a_3	14° 47' 18.84" N	120° 16' 12.12" E	KY069371	KY069490	KY069587		KY069678	
		MEFGL_45_11a_4	14° 47' 18.84" N	120° 16' 12.12" E	KY069372	KY069491	KY069588		KY069679	
	Mindoro	ZMBunid1258**	12° 37' 07.20" N	121° 24' 06.00" E	KY069435					
	Palawan	ZMBunid839**	10° 48' 52.02" N	119° 30' 42.00" E	KY069441					

			ZMBunid840**	10° 44' 57.00" N	119° 30' 09.00" E	KY069442				
			ZMBunid841**	09° 41' 19.98" N	118° 27' 15.00" E	KY069443				
			ZMBunid842**	09° 46' 36.00" N	118° 39' 55.02" E	KY069444				
			ZMBunid843**	09° 41' 19.98" N	118° 27' 14.28" E	KY069445				
			ZMBunid963**	09° 55' 33.00" N	118° 38' 19.80" E	KY069446				
			ZMBunid1259**	12° 01' 60.00" N	119° 59' 31.20" E	KY069436				
			ZMBunid1267**	12° 15' 25.80" N	119° 59' 46.20" E	KY069437				
		Samar	ZMB29547_1	11° 47' 07.02" N	124° 55' 36.00" E	KY069426				
			ZMB29547_2	11° 47' 07.02" N	124° 55' 36.00" E	KY069427				
			ZMB29548	12° 01' 60.00" N	124° 55' 30.00" E	KY069428	KY069545			
	Taiwan		ZMBunid1283**	22° 12' 00.96" N	120° 52' 42.06" E	KY069440				
	Seychelles		MEFGL_CT_Sey01	04° 37' 41.20" S	055° 25' 34.50" E	KY069374	KY069493	KY069590		KY069749
			MEFGL_CT_Sey02	04° 37' 41.20" S	055° 25' 34.50" E	KY069375	KY069494	KY069591		KY069750
			MEFGL_CT_Sey03	04° 37' 02.70" S	055° 25' 45.00" E	KY069376	KY069495	KY069592		KY069751
			MEFGL_CT_Sey04	04° 37' 02.70" S	055° 25' 45.00" E	KY069377	KY069496	KY069593		KY069752
			MEFGL_CT_Sey05	04° 37' 02.70" S	055° 25' 45.00" E	KY069378	KY069497	KY069594		KY069753
			MEFGL_CT_Sey06	04° 37' 02.70" S	055° 25' 45.00" E	KY069379	KY069498	KY069595		
	Mascarene Islands	Mauritius	OUMNH.ZC-2010-22-0001	23° 23' 00.00" S	57° 25' 00.00" E	KY069380	KY069499	KY069596		KY069754
			OUMNH.ZC-2010-22-0007	20° 06' 40.00" S	57° 32' 33.00" E	KY069382	KY069501	KY069598		
		Rodrigues	OUMNH.ZC-2010-22-0003	19° 42' 00.00" S	63° 24' 30.00" E	KY069381	KY069500	KY069597		KY069755
		Reunion	ZMBunid1252**	20° 55' 10.50" S	055° 36' 14.88" E	KY069434				
	Australia*		GUC571	19° 14' 24.00" S	146° 29' 24.00" E	DQ478561				
			GUCCY1	20° 20' 00.00" S	148° 39' 00.00" E	DQ478562	DQ478478.1			
	Japan*	Okinawa	GUC895	26° 45' 00.00" N	128° 18' 00.00" E	DQ478560				
	New Caledonia*		GUC868	20° 20' 02.00" S	164° 18' 51.00" E	DQ478558				
			GUC871	22° 23' 11.00" S	166° 55' 03.00" E	DQ478559	DQ478477.1			
	Sri Lanka*		FB-2311SL	____________	____________	AY708118				
	Vanuatu*		GUC721	14° 24' 00.00" S	145° 16' 12.00" E	DQ478563				
Caridina villadolidi	Indonesia	Sulawesi	ZMB29350	3° 40' 59.70" S	122° 15' 17.88" E	KY436221				
	Philippines	Mindoro	ZMBunid968**	13° 13' 51.00" N	120° 34' 54.00" E	KY436220				
			ZMBunid1227**	12° 27' 51.00" N	121° 02' 07.80" E	KY436222				
			ZMBunid1228**	12° 27' 51.00" N	121° 02' 07.80" E	KY436223				
	Taiwan		ZMBunid1278**	21° 59' 18.30" N	120° 49' 57.36" E	KY436224				

Caridina cf. opaensis	Indonesia	Sulawesi	ZMB29014_3	04° 23' 17.04" S	122° 35' 33.18" E	KY069328	KY069447		KY069717
			ZMB29014_4	04° 23' 17.04" S	122° 35' 33.18" E	KY069329	KY069448		KY069718

Notes: Countries marked with * had their samples taken from GenBank. With exception of them, these samples were taken from the Museum für Naturkunde Berlin (voucher numbers started in ZMB), Museum Victoria (voucher numbers started in CT_Ar), Oxford University Museum of Natural History (voucher numbers started in OUMNH) and collected samples deposited in the Molecular Ecology and Fisheries Genetics Laboratory in Bangor University (all others). Samples marked with ** had no repository number at the moment of the submission of this paper, but they are deposited in the Museum für Naturkunde Berlin. Samples that have two GenBank accession numbers are heterozygotes and due to differential gaps between alleles, they were deposited twice.

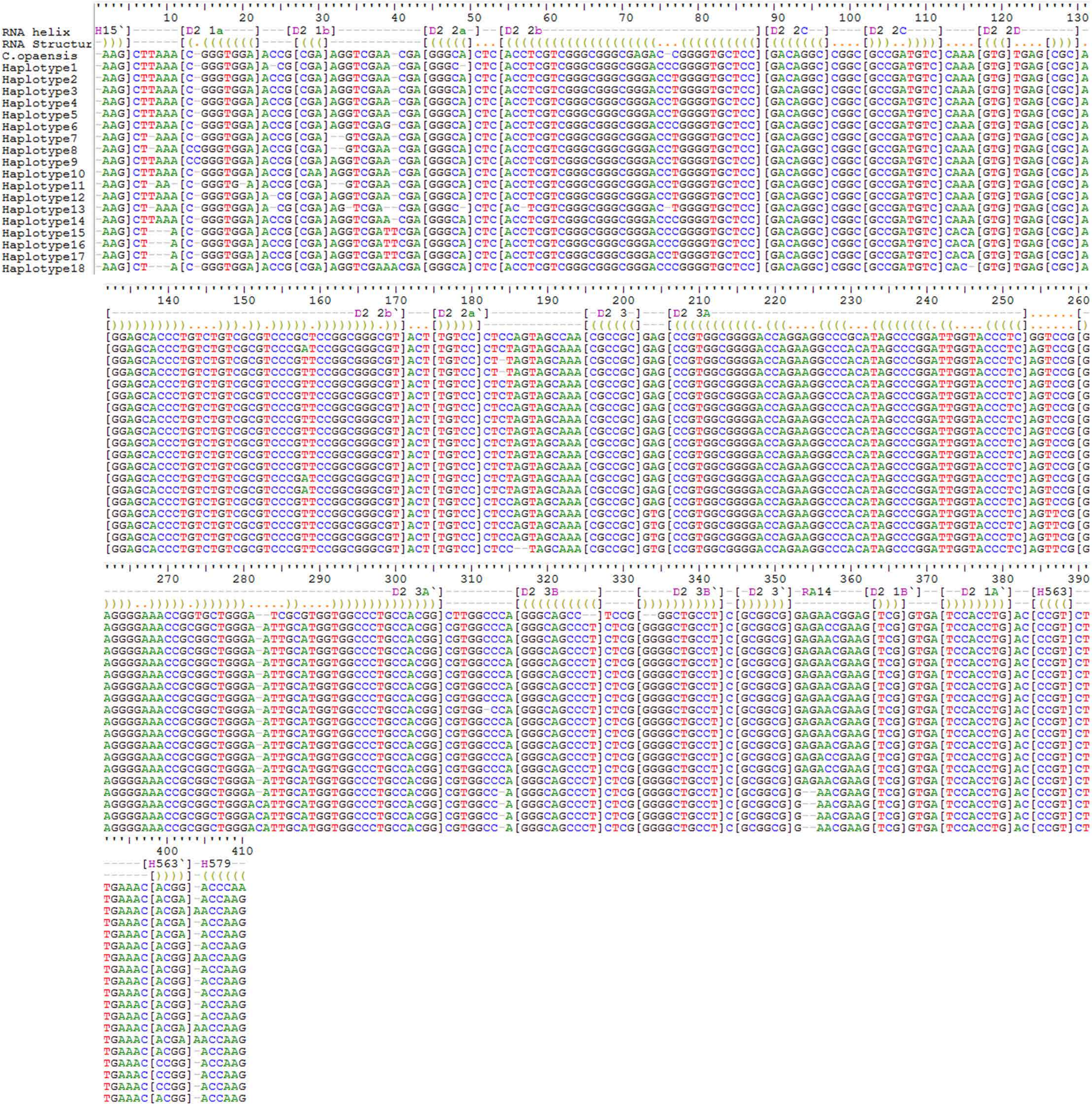

Supplementary Figure S1: 28S alignment through secondary structure for the 18 haplotypes represented in the network (Supplementary Figure S4)

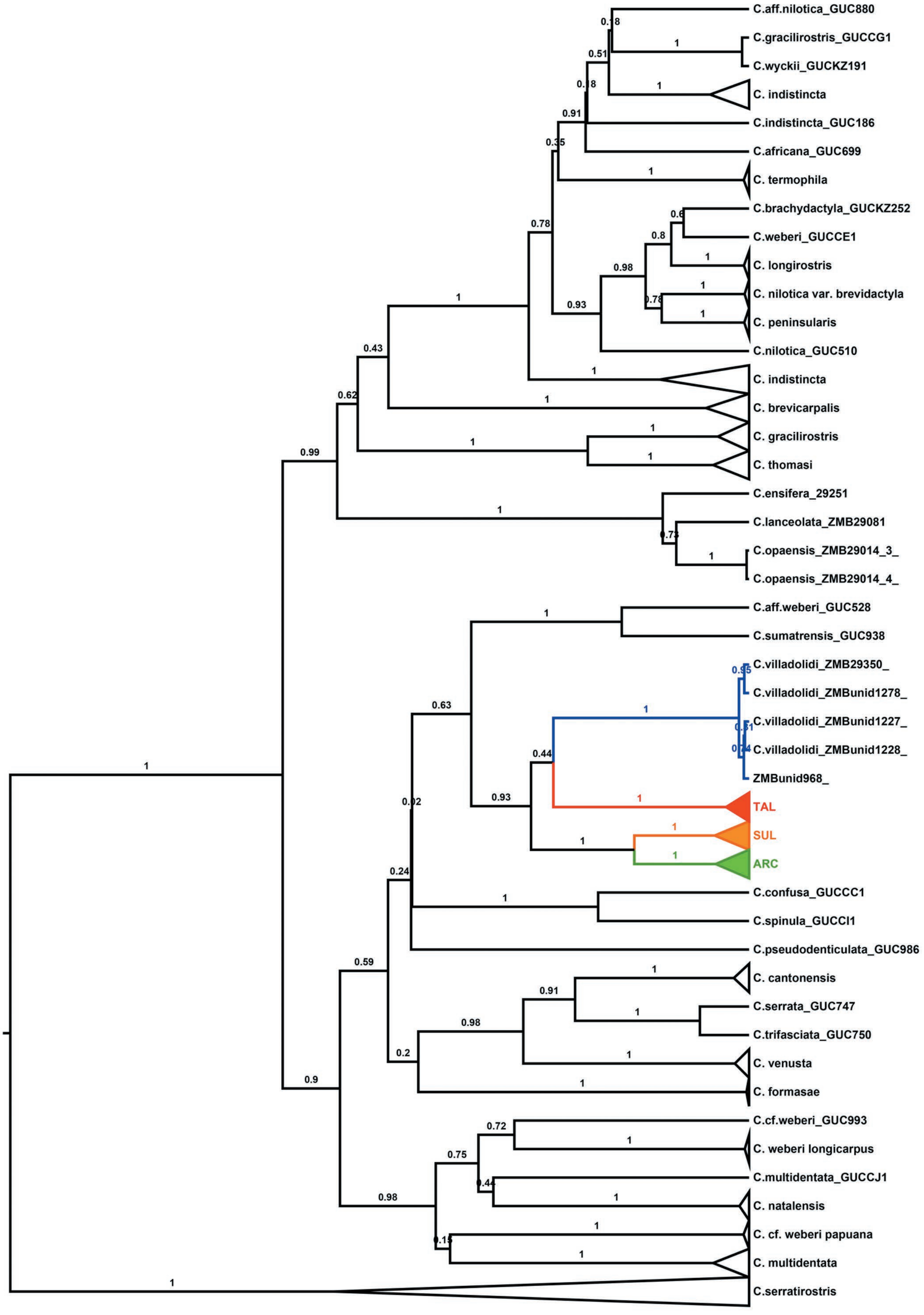

Supplementary Figure S2: Multispecies mitochondrial 16S Bayesian Caridina tree. The values on the branches represent the posterior probability of that clade. *C. typus* complex is coloured: in blue, *C. villadolidi*; in red, TAL; in orange, SUL; and in green, ARC.

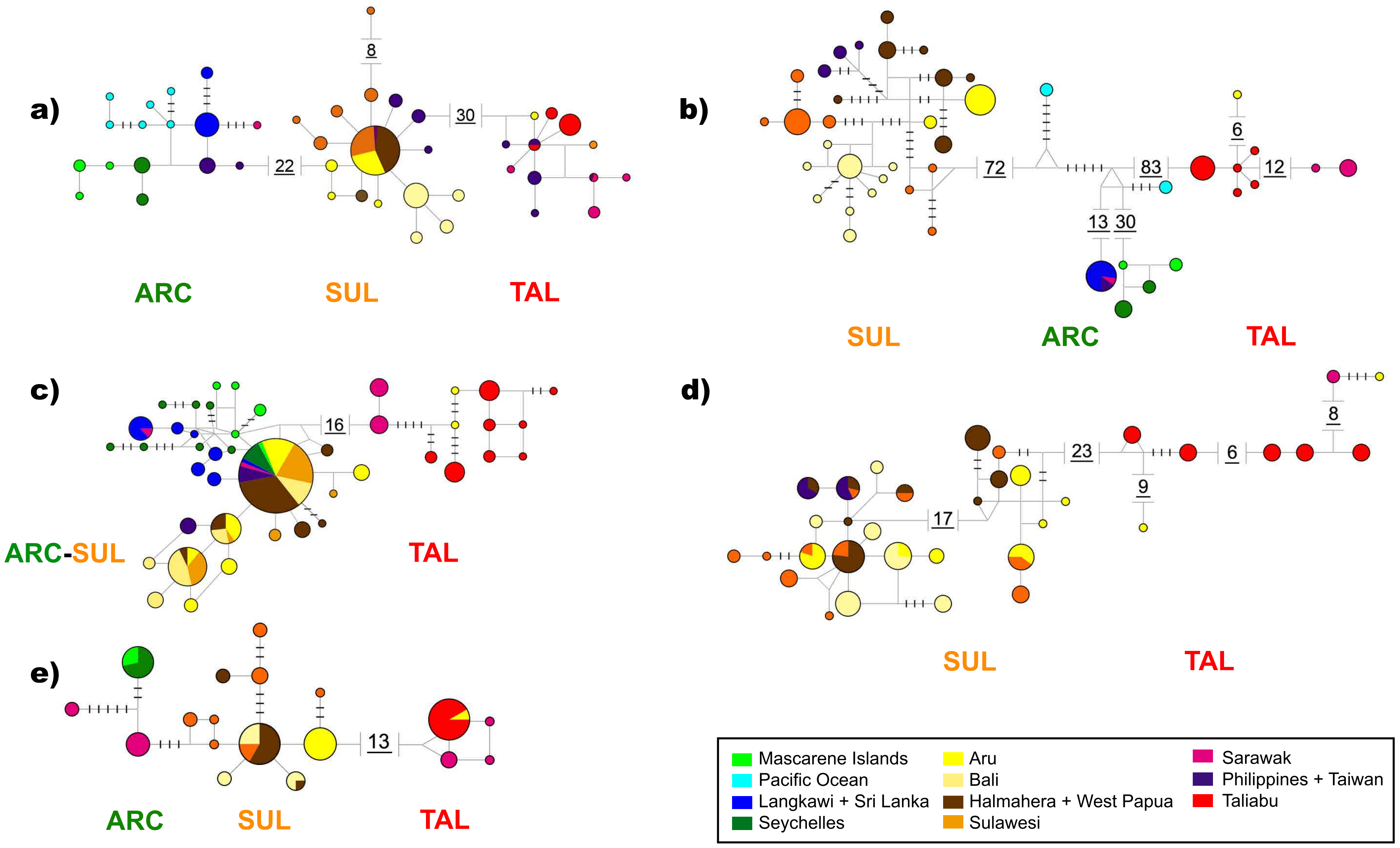

Supplementary Figure S3: Haplotype networks for all five marker 16S (a), COI (b), Ct33 (c), Ct51 (d), and 28S (e). A single substitution step is represented by a single line connecting two haplotypes. Any number of steps between two and five is represented by the number of dashes on the line connecting two haplotypes. Any distance higher than five steps is represented by the written number of steps. Non-sampled intermediary haplotypes are represented by empty vertices connecting three or more haplotypes. Each colour represents a region according to the legend.

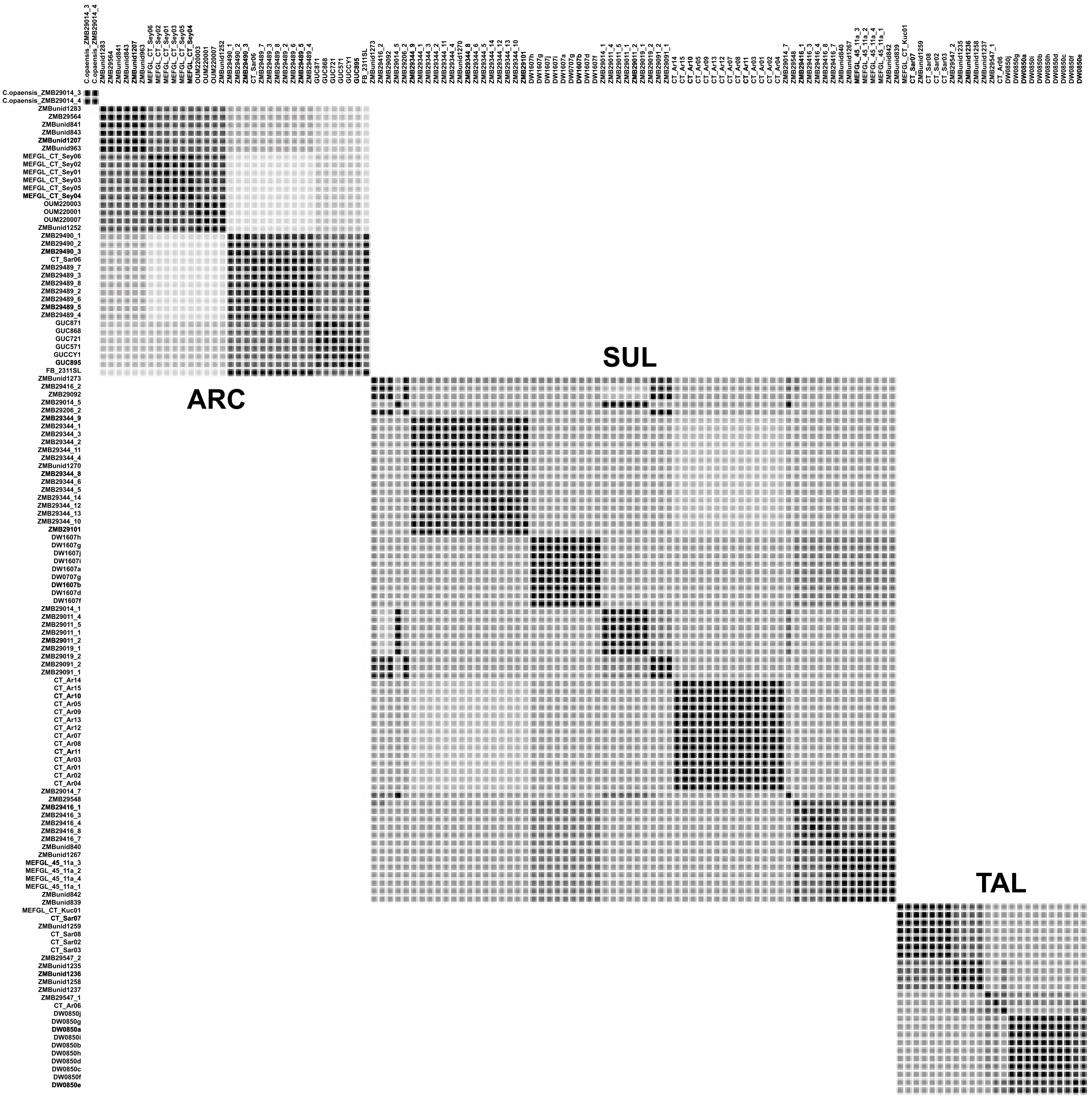

Supplementary Figure S4: STACEY matrix. The squares show the posterior probability of each pair of samples belonging to the same cluster. Black means 100% probability and white means 0% probability. Scale of grey indicates intermediary values.

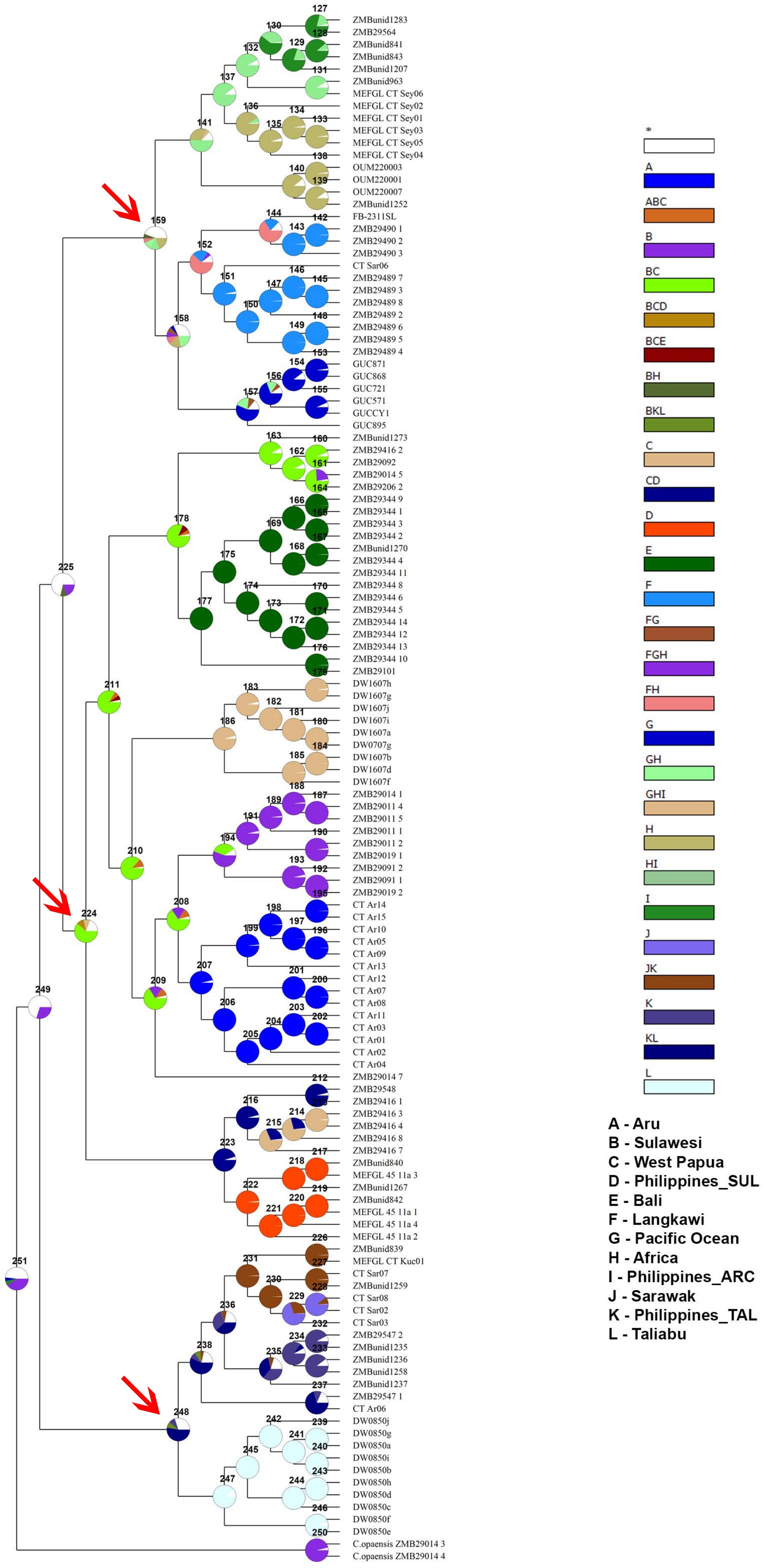

Supplementary Figure S5: RASP's BayArea full result. Each node is represented by a pie chart of relative probabilities between possible ancestral ranges. Each colour in those charts represents a combination of areas according to the legend. The node id is the number above the pie chart. Nodes marked by a red arrow represent the ancestors of each main clade.

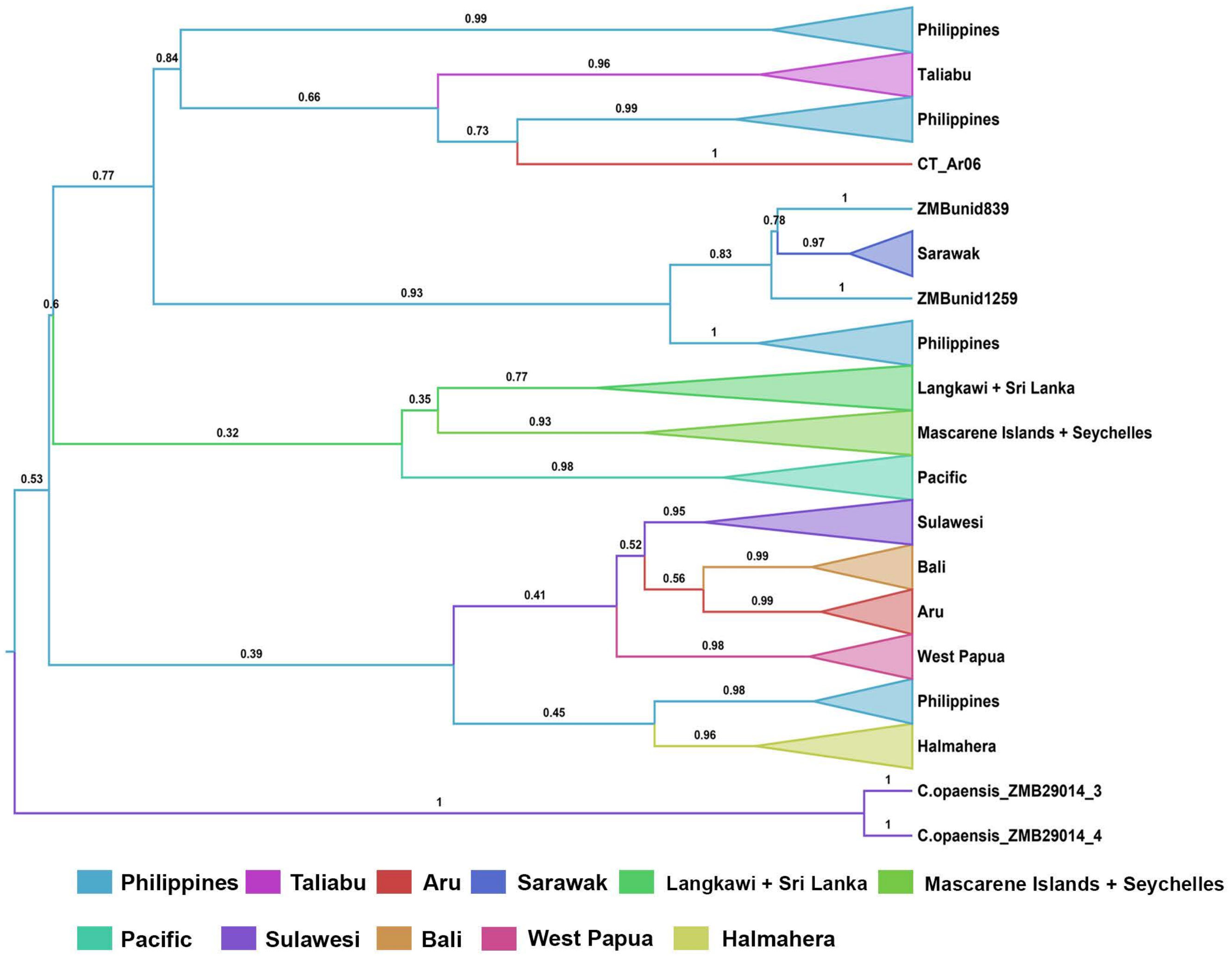

Supplementary Figure S6: BEAST discrete phylogeography result with collapsed branches per range. Each colour represents an area, including the colours applied on internal branches. The numbers on the branches represent the posterior probability of a given locality being the ancestor range, given that each terminal has a *pp* of 1.0 for its location.

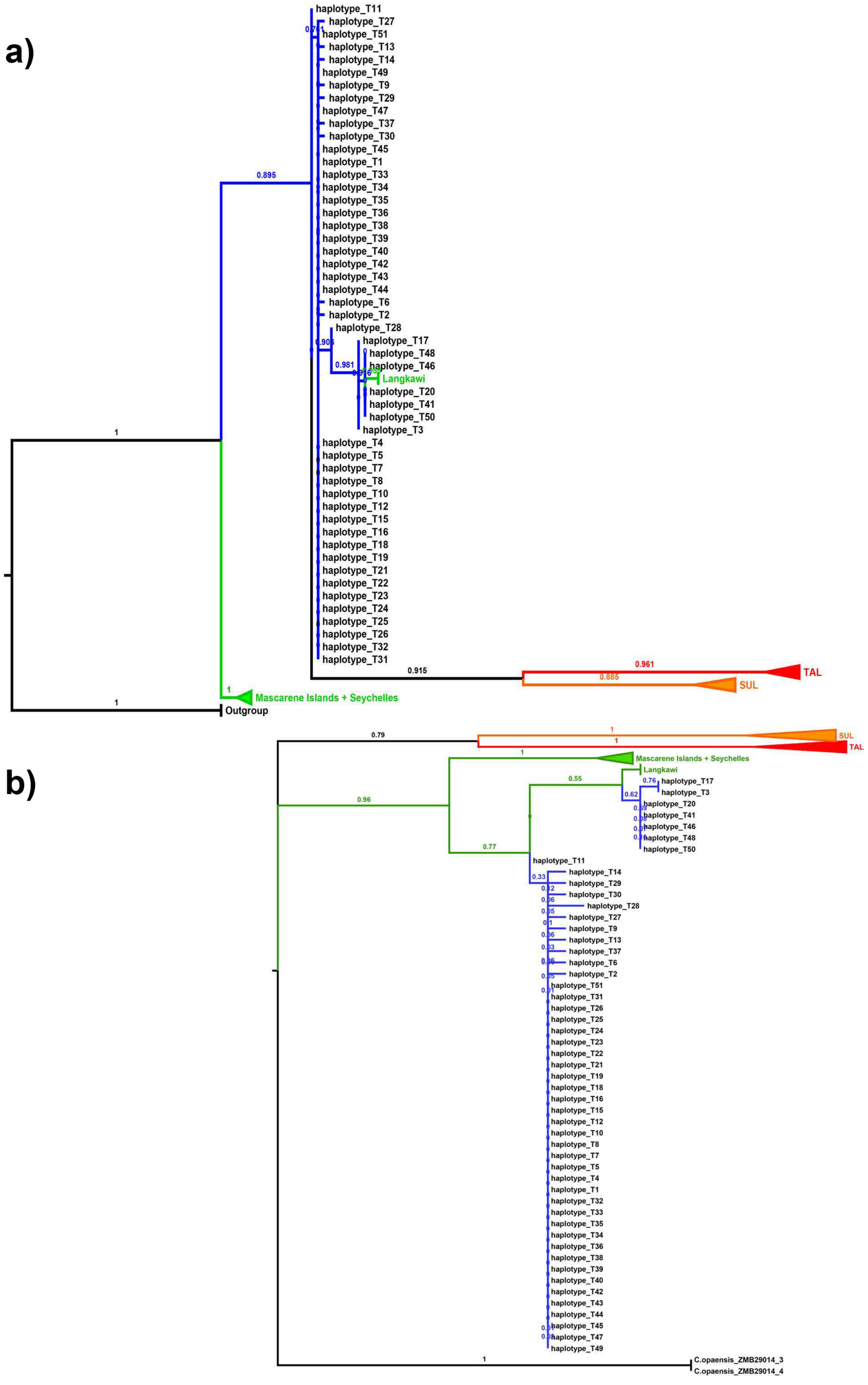

Supplementary Figure S7: Partial COI (167 nucleotide) maximum likelihood (a) and neighbour-joining (b) tree including Fujita et al.'s[1] sequences. In (a), the tree was build in PhyML[2] under a HKY model with invariable sites and gamma parameters estimated; in (b), the tree was build in MEGA6[3] under a Kimura 2-parameter model with gamma distributed rates. The numbers on the branches represent bootstrap values where 1 equals 100.

References

1. Fujita, J. et al. Comparative phylogeography to test for predictions of marine larval dispersal in three amphidromous shrimps. *Mar. Ecol. Prog. Ser.* **560**, 105–120 (2016).

2. Guindon, S. et al. New Algorithms and Methods to Estimate Maximum-Likelihood Phylogenies: Assessing the Performance of PhyML 3.0. *Syst. Biol.* **59**, 307–321 (2010).

3. Tamura, K., Stecher, G., Peterson, D., Filipski, A. & Kumar, S. MEGA6: Molecular Evolutionary Genetics Analysis Version 6.0. *Mol. Biol. Evol.* **30**, 2725–2729 (2013).
